# Interleukin-6 inhibition in the treatment of autoinflammatory diseases

**DOI:** 10.3389/fimmu.2022.956795

**Published:** 2022-07-26

**Authors:** Tomohiro Koga, Atsushi Kawakami

**Affiliations:** ^1^ Department of Immunology and Rheumatology, Division of Advanced Preventive Medical Sciences, Nagasaki University Graduate School of Biomedical Sciences, Nagasaki, Japan; ^2^ Center for Bioinformatics and Molecular Medicine, Nagasaki University Graduate School of Biomedical Sciences, Nagasaki, Japan

**Keywords:** amyloidosis, autoinflammatory diseases, familial mediterranean fever, inflammasome, interleukin-6

## Abstract

Autoinflammatory diseases are characterized by abnormalities that prevent innate immune cells from producing autoantibodies. While interleukin (IL)-6 is not directly associated with inflammasomes, like IL-1β or IL-18, it plays an important role in the pathogenesis of autoinflammatory diseases. Studies of autoinflammatory diseases, such as familial Mediterranean fever, cryopyrin-associated periodic syndrome, and tumor necrosis factor receptor-associated periodic syndrome, have shown IL-6 to be a promising therapeutic target. It has also been suggested that inhibition of IL-6 may have a therapeutic effect on amyloidosis, which is frequently associated with these chronic inflammatory diseases. In this study, we discuss the most recent research on the role of IL-6 in autoinflammatory diseases and its potential as a therapeutic target in their treatment.

## Introduction

Autoinflammatory diseases (AIDs) are characterized by systemic inflammation with recurrent fever and serositis. Clinical presentation may include arthralgia, skin rash, and abdominal pain (1). AIDs result from abnormalities of the innate immune system. Kastner defines the features of autoinflammation as 1) inflammatory findings with no apparent triggers; 2) no evidence of autoantibodies or autoreactive T cells; 3) inborn errors of innate immunity. AIDs that meet these criteria include familial Mediterranean fever (FMF), cryopyrin-associated periodic syndrome (CAPS), and tumor necrosis factor (TNF) receptor-associated periodic syndrome (TRAPS) (2).

Interleukin-6 (IL-6) is a major proinflammatory cytokine produced by various cells, including monocytes, macrophages, T cells, B cells, and fibroblasts (3). IL-6 receptors may be membrane-bound or soluble secreted receptors found in human serum. Both of these types of IL-6 receptors transduce intracellular signals through interactions with glycoprotein 130 (gp130). This takes place *via* two major pathways: the Janus kinase-signal transducers and activators of transcription (JAK-STAT) pathway and the mitogen-activated protein kinase pathway (4, 5).

Drugs that target IL-6 are biological agents, such as tocilizumab (TCZ), sarilumab, and siltuximab, which produce antiinflammatory effects by inhibiting IL-6. IL-6 inhibitors have been found efficacious in the treatment of idiopathic multicentric Castleman’s disease (6), rheumatoid arthritis (7), juvenile idiopathic arthritis, adult-onset Still’s disease (8), Takayasu arteritis (9), giant cell arteritis (10), cytokine release syndrome associated with tumor-specific T cell infusion therapy (11), and coronavirus 2019 (COVID-19) (12).

The pathogenesis of AIDs involves the abnormal activation of inflammasomes, which results in the overproduction of IL-1β and IL-18. These, in turn, stimulate the production of inflammatory cytokines such as IL-6 and TNF-α and contribute to the activation of immune cells such as macrophages and T cells (13). Therefore, therapies that inhibit IL-6 have been attracting attention as potential treatments for both the above-mentioned diseases and for AIDs, such as FM. In this review, we discuss the advantages and disadvantages of such therapies for the treatment of AIDs.

## The role of IL-6 in autoinflammatory diseases

Inflammation in AIDs results from the antigen-independent activation of immune cells. Many of these diseases present with recurrent fever; these are classed as periodic fever syndromes. Protein complexes called inflammasomes are thought to be central to these conditions. These can be activated by a variety of stimuli, including bacterial organisms, microbial toxins, foreign organisms, pathogen-associated molecular patterns (PAMPs), and damage-associated molecular patterns (DAMPs).

**Figure 1:**
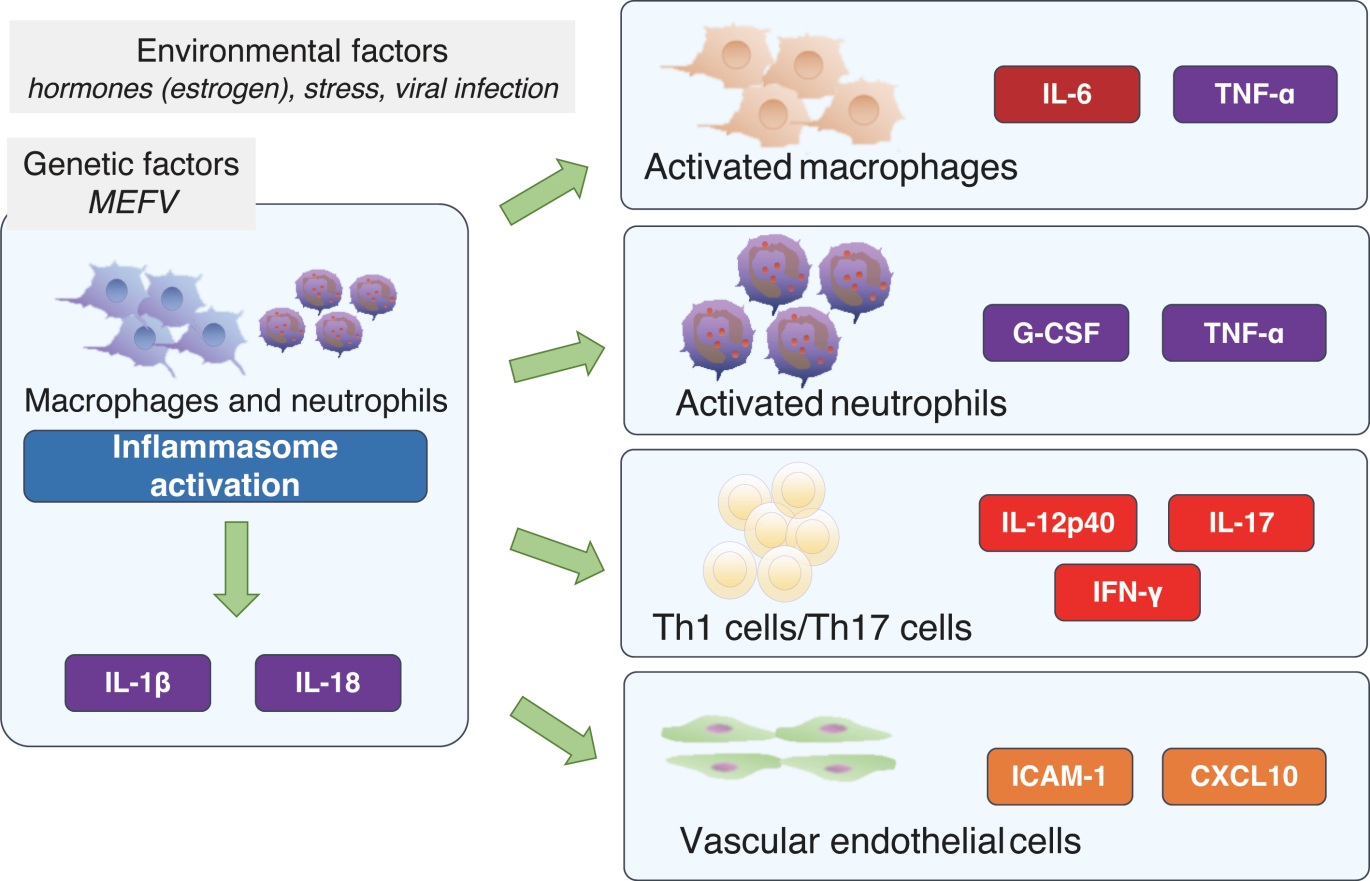
Cellular signals associated with inflammasome activation and proteins affected by autoinflammatory disease-related gene variants. ASC, apoptosis-associated speck-like protein containing a caspase recruitment domain; CAPS, cryopyrin-associated periodic syndrome; DAMPs, damage-associated molecular patterns; FMF, familial Mediterranean fever; IL, interleukin; IL-1R, interleukin-1 beta receptor; NLRP3, nucleotide-binding oligomerization domain-like receptor protein 3; PAMPs, pathogen-associated molecular patterns; SAA, serum amyloid A; TNF, tumor necrosis factor; TNFR, tumor necrosis factor receptor; TRAPS, TNF receptor-associated periodic syndrome.

Among the proteins of which inflammasomes are composed are pyrin, which is known to be dysfunctional in FMF; nucleotide-binding oligomerization domain-like receptor protein 3 (NLRP3), which is known to be dysfunctional in CAPS; and apoptosis-associated speck-like protein containing a caspase recruitment domain (ASC), which activates caspase 1. Activated caspase 1 cleaves IL-1β precursors to produce mature IL-1β. In the priming phase, the production of IL-1β depends on the induction of IL-1β precursors. This is brought about by the activation of inflammatory transcription factors such as nuclear factor kappa B (NF-κB) *via* toll-like receptors (TLRs), IL-1 receptors, and TNF receptors (which are dysfunctional in TRAPS). The IL-6 cytokine is produced both during this priming phase and in the process of mature IL-1β production. IL-6 is a potent inducer of serum amyloid A (SAA), which contributes to a positive feedback loop in inflammasomes by acting as a ligand. The relationship between mutations observed in AIDs and the various cytokines associated with inflammasome activation is shown in **Figure 1**.

### The role of IL-6 in familial mediterranean fever

IL-6 has been shown to be extremely elevated during FMF attacks (14), reflecting inflammasome hyperactivity due to abnormal pyrin function. On the other hand, elevated IL-6 is not detected during the intermittent phase of attacks (14), suggesting that it is not a marker of subclinical inflammation. The presence of cases of FMF that have responded to IL-6 inhibitors, which will be discussed in more detail below, may provide evidence that IL-6 is important in the pathogenesis of FMF.

### The role of IL-6 in cryopyrin-associated periodic syndrome

CAPS is caused by gain-of-function mutations in the *NLRP3* gene, which encodes cryopyrin, resulting in increased IL-1 secretion; clinical manifestations of CAPS include cold-induced episodes, urticaria-like rash, sensorineural hearing loss, chronic meningitis, myalgia and arthritis (15). It is classified according to severity as one of three diseases: the mildest, familial cold autoinflammatory syndrome (FCAS); the moderate Muckle-Wells syndrome (MWS); and the most severe, chronic infantile neurocutaneous and joint syndrome (CINCA), also known as neonatal-onset multisystem inflammatory disease (NOMID) (15). The importance of IL-6 in IL-1-mediated diseases such as CAPS is suggested by the fact that IL-6 transcription is induced by IL-1β. Indeed, higher levels of circulating IL-6 have been detected in CAPS patients compared to healthy controls, and IL-1 blockade in CAPS has been reported to reduce serum levels of IL-6 (16).

### The role of IL-6 in TNF receptor-associated periodic syndrome

TRAPS is a rare autosomal dominant inherited autoinflammatory disorder characterized by recurrent episodes of fever, myalgia, arthralgia, migrating erysipelas, and serositis. The disease is associated with missense mutations in TNF receptor superfamily 1A (TNFRSF1A) (17), and if untreated, attacks may last for days to weeks. Inadequate control of inflammation may increase the morbidity of amyloidosis. Both IL-6 and IL-8 have been reported to be elevated in TRAPS (18), and tocilizumab, an IL-6 receptor antibody, has been reported to be useful as a treatment for patients with TRAPS who have been unsuccessfully treated with TNF inhibitors and IL-1 inhibitors (19–21).

## The pathological significance of IL-6 in familial mediterranean fever

FMF, the most common AID, is characterized by recurrent episodes of fever due to arthritis and serositis (22). The etiology of FMF is polymorphisms and mutations in the Mediterranean fever (MEFV) gene that encodes pyrin (23). Since pyrin is involved in the regulation of the NLRP3 inflammasome, pyrin dysfunction leads to inflammation *via* the increased production of proinflammatory cytokines, including IL-1β and IL-18 (24). These cytokines activate the NF-κB signaling pathway, which increases TNF-α and IL-6 release (25).

To verify the importance of IL-6 in FMF, we performed a comprehensive analysis of the serum cytokine arrays of 75 Japanese FMF patients and 40 healthy controls. We used the random forest method to rank cytokine importance and found IL-6 to be the most important cytokine in the discrimination between attack and nonattack phases of the illness, as well as between FMF patients undergoing an attack and healthy subjects (26). Furthermore, the results of a multivariate classification algorithm with logistic regression analysis showed the combined measurement of IL-6, IL-18, and IL-17 to be the most accurate biomarker (sensitivity 89.2%, specificity 100%, accuracy 95.5%) of the attack phase in FMF patients and healthy subjects. The combined measurement of IL-6, G-CSF, IL-10, and IL-12p40 discriminated between the attack and nonattack phases in FMF patients with the highest accuracy (sensitivity 75.0%, specificity 87.9%, accuracy 84.0%) (26).

**Figure 2:**
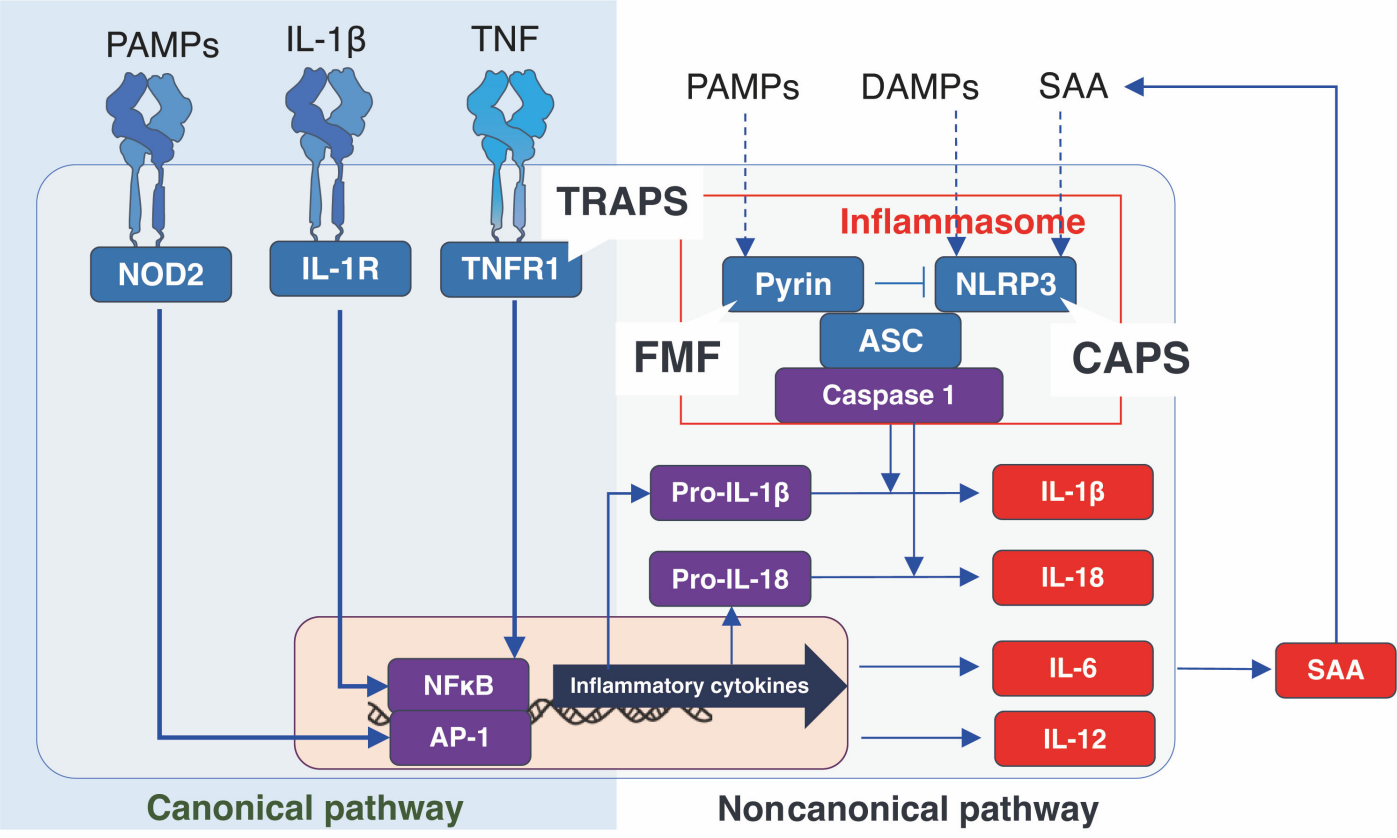
The cytokine network in familial Mediterranean fever. CXCL10: interferon gamma-inducible protein 10, G-CSF, granulocyte-colony stimulating factor; ICAM-1, intercellular adhesion molecule 1; IFN-γ, interferon-γ; IL, interleukin; TNF, tumor necrosis factor.

The cytokine network in FMF is shown in **Figure 2**. The FMF attacks can be triggered by emotional, physiological, or physical stress, viral infection, or even menstruation (27, 28). Accordingly, in addition to genetic factors, environmental factors can also cause the activation of inflammasomes, which, in turn, activates a variety of cells involved in the immune response. With regard to hormones, estrogen has been suggested to have an impact on the pathogenesis of FMF. It has been reported that some female FMF patients experience fever attacks that coincide with the menstrual cycle (29). In addition, estrogen has been shown to exhibit anti-inflammatory effects by suppressing the production of IL-1β (30), and a recent observational study of Japanese FMF patients showed that the efficacy of colchicine was higher in females than in males (31). For example, activated macrophages produce IL-6 and TNF-α, and activated neutrophils produce G-CSF and TNF-α. Furthermore, T cell activation induces the production of IL-12p40 and IFN-γ, which are Th1-related cytokines, and IL-17, a Th17-related cytokine. It is thought that vascular endothelial cells may also be activated, leading to the production of ICAM-1 and CXCL10 (26).

The above findings suggest that IL-6 is a useful biomarker for FMF and that TCZ, which specifically inhibits IL-6 signaling, is a useful therapeutic agent for FMF.

## The pros and cons of IL-6 inhibitors in the treatment of familial mediterranean fever

The objectives of FMF treatment are to control chronic inflammation, improve quality of life, and prevent secondary amyloidosis, which can cause irreversible organ damage. However, in daily practice, FMF therapy is often introduced to prevent febrile attacks and normalize the levels of acute-phase reactants such as C-reactive protein (CRP). The first choice of treatment is colchicine, which is effective in preventing FMF attacks and the development of secondary amyloidosis (32). This is the recommended treatment for all FMF patients regardless of the frequency and intensity of attacks (33); yet, around 10% of FMF patients are unresponsive or resistant to colchicine (34, 35).

Indeed, canakinumab, an anti-IL-1 therapy, has proven antiinflammatory effects, demonstrated in both clinical and laboratory findings, in patients with FMF who are resistant or intolerant to colchicine (29, 36–38). In addition, there are indications that IL-1β inhibitors can have therapeutic effects on amyloidosis (39) but, as yet, there is insufficient evidence for this.

Notably, IL-6 inhibitors can dramatically reduce the production of serum amyloid A (SAA) protein and may have greater efficacy in the treatment of amyloidosis (40). Studies have shown that IL-6 plays an important role in the synergistic induction of the human SAA gene and that IL-6 inhibition is necessary for the complete suppression of SAA production (40). These findings may suggest that IL-6 inhibitors have greater potential than other biological agents to directly control amyloidosis.

Recently, there have been several case reports describing successful treatment with IL-6 inhibitors (41). A case reported by Fujikawa et al. presented with symptoms of adult Still’s disease, including hyperferritinemia and rash, and was treated with TCZ (42). A case study by Hamanoue et al. described a patient with FMF and AA amyloidosis initially treated unsuccessfully with colchicine. Subsequent treatment with TCZ treatment resulted in reductions in amyloid deposition (43). A patient described by Umeda et al. developed FMF with myositis symptoms and was treated with colchicine. This had no effect but, again, greater improvements were observed with TCZ treatment, which controlled fever attacks and muscle symptoms (44). Similarly, Honda et al. reported a case of protracted febrile myalgia syndrome, a subtype of FMF, with a variant of M694I in exon 10. This was successfully treated with tocilizumab (45). A case report from Turkey, where the prevalence of FMF is high, demonstrated similar results (46). More recently, a retrospective study examined the frequency of recurrent attacks of FMF before and after TCZ administration in 15 patients treated with TCZ for amyloidosis. Of the 15, one patient showed no response to the treatment, six experienced a reduction in the frequency of attacks, and eight patients demonstrated a complete cessation of attacks (47). Therefore, TCZ may be recommended for FMF patients in the early stages of AA amyloidosis and for those unresponsive to colchicine to prevent AA amyloidosis from developing.

A placebo-controlled randomized controlled trial was conducted to evaluate the efficacy and safety of TCZ in Japanese FMF patients unresponsive or intolerant to colchicine. While the primary goal of reduction in the number of fever attacks was not achieved, TCZ was found to effectively reduce febrile attacks and other FMF symptoms and showed stable outcomes after long-term treatment (48).

However, this latter study is among the few clinical trials, and data on the use of IL-6-targeted agents in FMF patients are limited. Further clinical trials are needed to evaluate the efficacy and safety of anti-IL-6 reagents.

## Potential therapeutic targets and issues with IL-6 inhibitors in autoinflammatory diseases

**Table 1** shows recently completed or ongoing clinical trials for AIDs targeting the IL-6 pathway. A phase 2 clinical trial of FMF with tocilizumab has been completed in Germany (NCT03446209), but there is no description of CAPS or TRAPS. There are scattered reports demonstrating the efficacy of tocilizumab in FMF and other AIDs such as TRAPS. TRAPS is an autosomal dominant AID associated with mutations in the TNFRSF1A gene on chromosome 12p13. It is characterized by fever, abdominal pain, myalgia, joint pain or arthritis, and rash. Research has found that the treatment of TRAPS with TCZ can halt acute attacks and prevent further attacks for around 6 months (19). TCZ has been found effective in TRAPS patients who had previously failed to respond to etanercept and infliximab (both TNF-α inhibitors) (20). A case report from Japan found TCZ therapy effective in a 30-year-old female patient with TRAPS who was refractory to etanercept (21). These results suggest that IL-6 inhibition may be a viable therapy for refractory TRAPS patients who have not responded to TNF inhibition.

**Table 1:** Search results for tocilizumabHereditary Autoinflammatory Diseases in Clinicaltrials.gov.

Study Title	Status	Conditions	Interventions	Locations
Tocilizumab for the Treatment of Familial Mediterranean Fever	Completed	Familial Mediterranean Fever	Drug: Tocilizumab Infusion RoAcemtra (EU) Drug: 0.9% physiological saline	1. Universitätsklinikum Köln, Klinik I für Innere Medizin Cologne, NRW, Germany 2. Charité Universitätsmedizin Berlin, Klinik für Rheumatologie und Klinische Immunologie, Abteilung -Neue Therapien & Studien Berlin, Germany 3. University Hospital Tuebingen; Department of oncology, hematology, rheumatology, immunology and pulmology Tuebingen, Germany
Tocilizumab for the Treatment of Refractory Behcet’s Uveitis	Terminated	Behcet Syndrome Uveitis	Biological: Tocilizumab (TCZ)	Peking Union Medical College Hospital Beijing, Beijing, China
Tocilizumab for the Treatment of Behcet’s Syndrome	Terminated	Behcet Syndrome	Drug: Tocilizumab	NYU Center for Musculoskeletal Care New York, New York, United States

In a case study of a 13-year-old patient with hyperimmunoglobulin D and periodic fever syndrome, the patient had failed to respond to treatment with colchicine, corticosteroids, etanercept, and anakinra but was successfully treated with TCZ (49). IL-6 inhibitors have also been proposed as a treatment option for rare and severe AIDs such as vacuoles, E1 enzyme, X-linked, autoinflammatory, somatic (VEXAS) syndrome caused by somatic variants in the UBA1 gene (50, 51). The clinical features of VEXAS syndrome include high fever, polychondritis, macroangitis, skin rash, arthritis, thrombosis, scleritis, and serositis, and the prognosis is poor, despite trials with various immunosuppressive agents (52).

A potential issue with TCZ therapy is that IL-6 inhibition masks symptoms such as IL-6-dependent fever and laboratory findings such as raised CRP levels. This can delay the detection of infectious complications. Other disadvantages include the fact that hereditary AIDs can be more difficult to treat than inflammatory diseases such as rheumatoid arthritis, and there are safety and medical-economic issues with the long-term administration of IL-6 inhibitors.

## Conclusion

We have briefly reviewed the role of IL-6 in AIDs and considered the pros and cons of IL-6 inhibitors as a treatment for AIDs. Future research is needed to identify biomarkers, particularly genomic biomarkers, to develop precision IL-6-inhibiting treatments and gather high-quality evidence for the therapeutic potential of IL-6 inhibitors for AID treatment.

## Author contributions

AK reviewed and edited the manuscript. TK wrote the manuscript. All authors contributed to the article and approved the submitted version.

## Funding

This work was supported by the Japan Society for the Promotion of Science (JSPS) KAKENHI (grant number JP122K08544 to TK).

## Acknowledgments

The authors would like to thank our colleagues and staff at the Rheumatology Department of Nagasaki University Hospital for their support.

## Conflict of interest

The authors declare that the research was conducted in the absence of any commercial or financial relationships that could be construed as a potential conflict of interest.

## Publisher’s note

All claims expressed in this article are solely those of the authors and do not necessarily represent those of their affiliated organizations, or those of the publisher, the editors and the reviewers. Any product that may be evaluated in this article, or claim that may be made by its manufacturer, is not guaranteed or endorsed by the publisher.
